# Risk Factors That can Predict Antenatal Insulin Need in Gestational Diabetes

**DOI:** 10.4021/jocmr1515w

**Published:** 2013-08-05

**Authors:** Okan Bakiner, Emre Bozkirli, Kursat Ozsahin, Cagla Sariturk, Eda Ertorer

**Affiliations:** a Department of Endocrinology and Metabolism Diseases, Baskent University, Faculty of Medicine, Turkey; b Department of Family Medicine Baskent University, Faculty of Medicine, Turkey; c Department of Biostatistics, Baskent University, Faculty of Medicine, Turkey

**Keywords:** Diabetes mellitus, Gestational, Antenatal, Insulin, Risk factors

## Abstract

**Background:**

This study was undertaken to assess the association between insulin need in gestational diabetes mellitus (GDM) and clinical features and laboratory parameters. Factors that can predict insulin need are also identified.

**Methods:**

Cases with GDM were included retrospectively from records. Cases which failed to achieve target blood glucose levels with medical nutrition therapy (MNT) and need insulin treatment were recorded. Risk factors which can predict antenatal insulin treatment (AIT) were identified as follows; the presence of diabetes in a first degree relative, body mass index prior to pregnancy, number of parity, history of GDM, macrosomic baby delivery (> 4,000 g), age, gestational week at time of diagnosis, body mass index during diagnosis, weight gain untill diagnosis, mean systolic and diastolic blood pressure, HbA1C level during diagnosis, and fasting plasma glucose on diagnostic oral glucose tolerance test. Presence of a statistical significance between those patient features and AIT was assessed. Independent predictors for AIT were evaluated.

**Results:**

A total of 300 cases were recruited from records, 190 cases (63.3%) were followed only with MNT until delivery and 110 cases (36.7%) were initiated AIT. The association between AIT and patient factors like presence of diabetes in the pedigree, week of gestation at which GDM was diagnosed, BMI during diagnosis, HbA1C levels, and fasting plasma glucose during diagnosis was found (P = 0.03; 0.008; 0.049; 0.001 and 0.001respectively). Multivariant analysis showed that fasting plasma glucose levels during diagnosis and HbA1C levels were independent risk factors for AIT. Fasting plasma glucose values that can predict AIT were identified > 89.5 mg/dL with 72.7% sensitivity and 62.6% spesifity (P < 0.001). Positive predictive value was 73% (P < 0.001). Also, HbA1C levels that can predict AIT was found to be > 5.485% with 65.3% sensitivity and 66.7% spesifitiy(P < 0.001) with a positive predictive value 68% (P < 0.001).

**Conclusions:**

Independent predictors for AIT were found as fasting plasma glucose on OGTT and HbA1c levels during diagnosis in GDM. Cases with fasting plasma glucose ≥ 89.5 mg/dL or HbA1C ≥ 5.485% should be closely followed for AIT in specified centers.

## Introduction

Gestational diabetes mellitus (GDM) is a glucose metabolism disorder detected during pregnancy [1]. It is closely associated with perinatal outcome [2] and with the risk of developing type 2 diabetes in the future life of both fetus and the mother [3]. Maternal glucose levels demonstrate positive correlation with the risk of poor fetal development [2, 3].

Self-glucose monitoring is very useful to maintain glycemic control in GDM [4]. Target glucose levels are < 95 mg/dL at fasting, < 140 mg/dL at first hour, and < 120 mg/dL at second hour of starting a meal, according to the recommendations of the Fifth International Workshop Conference on GDM [5]. In the case of a women who do not achieve desired glucose levels with diet and exercise, drug therapy should be given to reduce glucose levels in order to guarantee good fetal development and to minimize neonatal complications [6]. The only drug approved so far by the Food and Drug Administration (FDA) for the use in diabetic pregnant women is insulin [7]. The need for insulin therapy might be a starting point for the characterization of patients with more severe GDM. It has been suggested that improved efficiency of health care delivery in GDM relies on improved risk stratification that would allow for triage to low- or higher-risk clinics [8]. Antenatal insulin treatment (AIT) is the most resource-intensive management component in GDM, a risk-prediction tool that identifies patients likely to need AIT would have theoretical utility. Some risk factors that indicate insulin therapy for glycemic control in pregnancies complicated by diabetes have been studied before. Diagnosis of diabetes at an early gestational age, fasting glucose levels, presence of obesity or a family history of diabetes or exacerbated fetal growth, are all associated with a more severe degree of glucose intolerance [9-12]. Similarly, evaluation of 100, 75 or 50 g oral glucose tolerance tests indicates the possible relationship between the severity of disorder and the number of abnormal blood glucose values (above the reference limits) [13, 14]. The role of glycated hemoglobin (HbA1C) in GDM remains controversial despite there are attempts for correlating this parameter with perinatal outcomes [15].

It seems possible to weigh different levels of glucose intolerance during pregnancy in terms of severity, but it remains uncertain above which threshold the risks for the pregnant woman and especially for the fetus reach a magnitude that requires more intensive and/or detailed monitoring [3]. The objective of the present study is to investigate the factors predicting insulin requirement during GDM and to determine the threshold at which AIT is needed.

## Method

### Patient selection

Pregnant women accepted to the Endocrinology and Metabolism outpatient clinic with the diagnosis of Gestational Diabetes between September 2011 and November 2012, were recruited retrospectively. Between 24th and 28th gestational week, GDM screening were performed with oral glucose tolerance test (OGTT) according to American Diabetes Association (ADA) 2011 guidelines [16]. Glucose levels were studied from antecubital venous samples just before and at first and second hour of 200 mL oral glucose solution intake containing 75 g glucose after 12 hours of fasting. Keeping in accordance with ADA gestational diabetes diagnosis criteria, the presence of at least one of the followings; fasting plasma glucose between 92 and126 mg/dL, plasma glucose > 180 mg/dL at the first hour and above 153 mg/dL at second hour during OGTT confirmed diagnosis [16].

The exclusion criteria were defined as having history of previous diabetes mellitus, being diagnosed with diabetes before 24th gestational week. Those who had fasting plasma glucose levels > 126 mg/dL, and/or who had HbA1C levels > 6.5% at diagnosis, smokers and multiple pregnancies were excluded, as well.

This study was approved by the Ethics Commite of Baskent University (Project no: KA12/278) and was supported by Baskent University Research Fund.

### Method

This was planned as a retrospective observational cohort study. The cases who needed AIT, due to inability in achieving target blood glucose levels with medical nutrition therapy (MNT) [5] were identified and registered. The MNT were prescribed by a registered dietician with respect to ADA 2011 standarts [16]. It was individualized in respect to pre-pregnancy body mass index (BMI) of the patients. Obesity was defined as BMI, calculated by pre-pregnancy weight (kg) divided by height (m, squared). Women with a BMI greater than 27 were given 25 kcal/kg of their actual body weight, those with BMI 20 - 26 were given 30 kcal/kg and those with BMI less than 20 were prescribed 35 kcal/kg. Physical activity for 30 min per day was recommended to all patients. Patients were educated to monitor their blood glucose levels. Capillary glucose measurements were performed by the patients at fasting, first and second postprandial hours. The decision for introduction of insulin was considered after evaluation of self-monitoring of blood glucose levels. Target maternal capillary glucose levels were below 96 mg/dL at fasting, < 140 mg/ dL at 1 h, and < 120 mg/dL at 2 h after starting the meal, according to Fifth International Workshop Conference on GDM [17]. Women who failed to achieve target fasting and postprandial glucose levels at the end of two-weeks of follow-up were prescribed insulin.

Patients characteristics from the records which could be decisive for antenatal insulin treatment were identified under two headlines.

#### Pregestational

The presence of diabetes mellitus among first degree relatives, BMI before gestation, number of parity, history of GDM in former pregnancies, history of giving birth to a macrosomic baby (> 4,000 g).

#### Gestational

Age, gestational week when diabetes was diagnosed, BMI during diagnosis, weight gain untill diagnosis, mean systolic and diastolic blood pressure during diagnosis, HbA1C and fasting plasma glucose levels atdiagnosis.

Statistical analyses of AIT and patient characteristics mentioned above was performed. Independent predictors and power of prediction between AIT and related patient characteristics through multivariant statistical analysis were inquired, as well.

### Assay methods

After overnight fasting, venous blood samples were withdrawn for determining glucose and HbA1c levels, % Hemoglobin A1c (DCCT/NGSP) values for human blood samples were obtained on a Roche Modular analyzer (Roche Diagnostics GmbH, Mannheim, Germany) using the Tina-quant Hemoglobin A1c Gen.3 reagent with the hemolysate application. Glucose levels were also mesured on a Roche Modular analyzer using glucose oxidase methodology.

### Statistical analysis

Statistical analysis was performed using the statistical package SPSS v 17.0. For each continuous variable, normality was checked by Kolmogorov Smirnov and Shapiro-Wilk tests and by histograms. Comparisons between gender or BMI were applied using Student T test or One Way ANOVA for normally distrubited data and Mann Whitney U test or Kruscall Wallis test were used for the data not normally distributed. A univariate analysis was done initially, and then clinical variables that were identified as significantly associated with the need for insulin use were included in a multivariate logistic regression. Values of P < 0.05 were considered as statistically significant.

## Results

Three-hundred of the 364 hyperglycemic pregnant women who met the inclusion criteria were included in the study. One-hundred ninety (63.3%) of cases were followed wih MNT alone and 110 (36.7%) of cases were given antenatal insulin therapy. Table 1 demonstates the comparison of pregestational and gestational factors between patients with MNT only and those with AIT.

**Table 1:** Comparison of Pregestational and Gestational Factors Between Patients With MNT Only and Those With AIT

	Factors	Patients with MNT only (n = 190)	Patients with AIT (n = 110)	P
Pregestational Factors	positive family history (n)	88 (46.3%)	64 (58.2)	0.031
	pregestational BMI (kg/m^2^)	26.2 ± 4.3	27.7 ± 5.5	0.122
	Parity	2 (1 - 8)	2 (1 - 8)	0.805
	history of GDM (n)	46 (24.2%)	32 (29%)	0.391
	macrosomic baby birth history (n)	11 (6.2%)	10 (%9.8)	0.190
Gestational Factors	age (year)	32.9 ± 4.6	32.9 ± 4.9	0.914
	diagnosis time (gestational week)	26.1 ± 5.1	27.6 ± 4.4	0.008
	BMI at diagnosis (kg/m^2^)	30.2 ± 4.6	31.6 ± 4.9	0.049
	weight gain until diagnosis (kg)	9.2 (0 - 16.2)	8.6 (2 - 18.3)	0.945
	systolic blood pressure during diagnosis (mmHg)	114.5 ± 8.8	113.6 ± 5.9	0.532
	diastolic blood pressure during diagnosis (mmHg)	69.2 ± 7.9	69.0 ± 7.9	0.089
	HbA1C during diagnosis (%)	5.3 ± 0.6	5.6 ± 0.6	0.001
	Fasting plasma glucose during OGTT (mg/dL)	87.5 ± 11.2	97.3 ± 12.1	0.0001

MNT: medical Nutrition Therapy; AIT: Antenatal insulin treatment; Mean ± Standard deviation, Median (Minimum-Maximum).

Considering about pregestational factors, the groups exhibited statistically significant difference only with the presence of positive family history of diabetes (P = 0.031). Diabetes was more prevalent among the family members of patients with AIT. Other factors; pregestational BMI, number of parity, history of GDM and giving birth to a macrosomic baby exhibited insignificant difference between the two groups (P = 0.12, P = 0.80, P = 0.39 and P = 0.19, respectively).

When gestational factors were evaluated, patients with AIT were found to be more obese at diagnosis, 30.2 ± 4.6 vs 31.6 ± 4.9 kg/m^2^ (P = 0.049). They also had higher HbA1C and fasting plasma glucose during OGTT (P = 0.001 and P = 0.0001, respectively). Diagnosis time of their diabetes was later than patients with MNT only, (P = 0.008). Other gestational factors; age, weight gain until diagnosis, systolic and diastolic blood pressures were statistically indifferent between the two groups (P > 0.05).

Multivariate analysis demonstrated that fasting glucose level during OGTT and HbA1C levels at diagnosis were significant predictors for insulin requirement in GDM (Table 2).

**Table 2:** Regression Analysis Demonstrating Fasting Glucose Level at OGTT and HbA1C at Diagnosis as Independent Predictors for Insulin Requirement in Gestational Diabetes Mellitus

	B	S.E.	Wald	df	P	Odd Ratio	95% C.I. for EXP(B)	
							Lower	Upper
positive family history	0.351	0.367	0.918	1	0.338	1.421	0.692	2.916
Diagnosis time (gestational week)	-0.046	0.044	1.115	1	0.291	0.955	0.876	1.040
BMI at diagnosis	0.026	0.038	0.458	1	0.499	1.026	0.952	1.106
HbA1C during diagnosis	0.680	0.316	4.635	1	0.031	1.974	1.063	3.666
Fasting plasma glucose at OGTT	0.062	0.016	14.947	1	0.0001	1.064	1.031	1.098
Constant	-9.682	2.665	13.203	1	0.0001	0.000		

a. Variable(s) entered on step 1 positive family history, time of diagnosis (gestational week), BMI at diagnosis, HbA1C at time of diagnosis, Fasting plasma glucose on OGTT.

**Figure 1:**
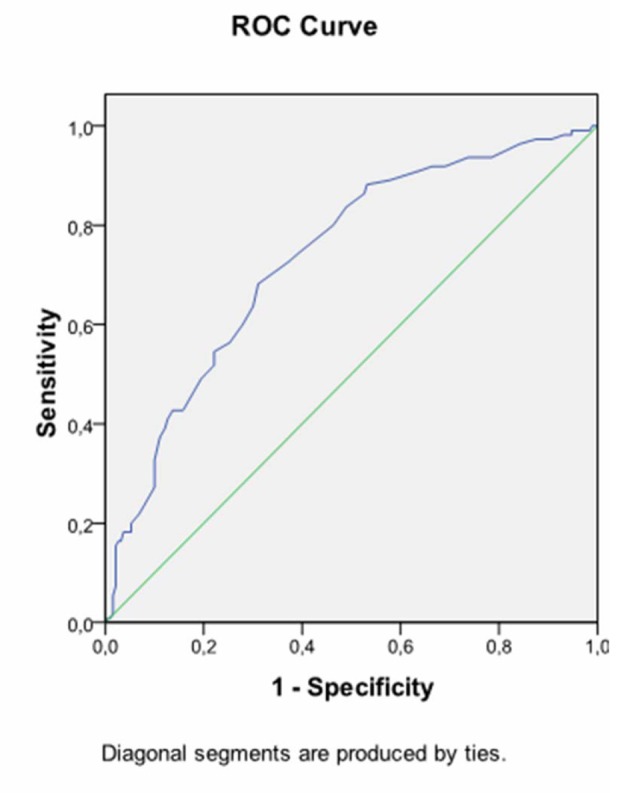
Receiver operating characteristic curve (ROC) exhibiting fasting plasma glucose during OGTT at prediction of insulin requirement. AUC 0.734, cutofflevel: 89.5 mg/dL, Sensitivity: 72.7% Specifity: 62.6%, P = 0.001.

**Figure 2:**
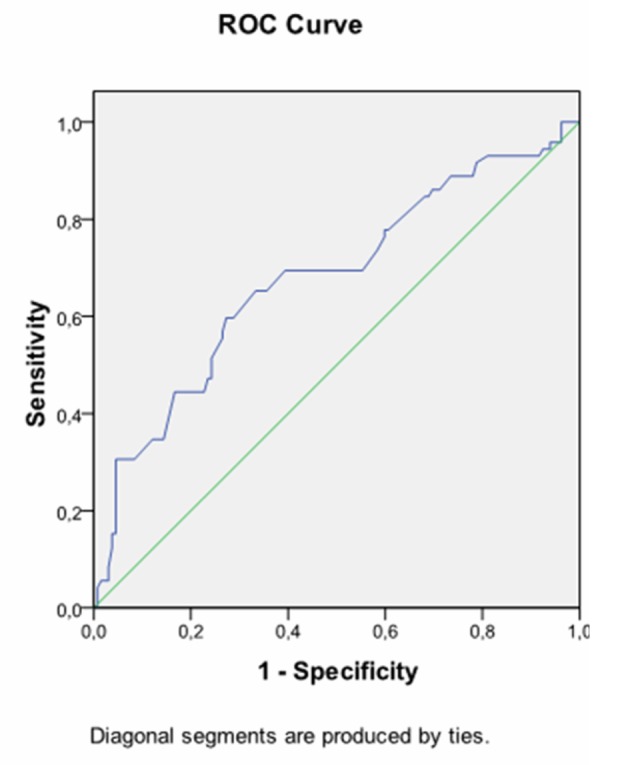
Receiver operating characteristic curve (ROC) demonstrating HbA1C at diagnosis in prediction of insulin requirement. AUC 0.677, cutofflevel: 5.485%, Sensitivity: 65.3%, Specifity: 66.7%, P = 0.001.

Receiver operating characteristic (ROC) curves were generated to determine cut-off values for fasting glucose and HbA1C levels at diagnosis to predict insulin requirement (Fig. 1, 2). Accordingly, 72.7% of women who had fasting glucose level equal to or higher than 89.5 mg/dL on OGTT required insulin treatment, whereas only 27.3% of women who had fasting glucose level below 89.5 mg/dL on OGTT were treated with insulin (P = 0.001). The positive predictive value of fasting glucose value ≥ 89.5 mg/dL on OGTT was 73%.

Considering about HbA1C, 51.6% of women who had HbA1C level equal to or higher than 5.485% at diagnosis required insulin treatment, whereas 22.1% of women who had HbA1C level below 5.485% at diagnosis were treated with insulin (P = 0.0001). The positive predictive value of HbA1C level ≥ 5.485% at diagnosis was 68%.

Odds ratio and 95% CI for fasting plasma glucose level during OGTT and HbA1C level at diagnosis are given in Table 3.

**Table 3:** Odds Ratio and 95% CI for Fasting Plasma Glucose During OGTT and HbA1C at Diagnosis

	Value	95% Confidence Interval	
		Lower	Upper
Odds ratio for fasting plasma glucose (89.5 mg/dL)	4.469	2.678	7.460
Odds ratio for HbA1C (5.485%)	3.760	2.053	6.887

## Discussion

Gestational diabetes mellitus is an important disorder which has poor outcomes for both fetus and the mother and early stabilisation of glucose levels plays a pivotal role in its management. As insulin is the only tool that is licenced for the management of glucose levels in pregnancy, it is crucial to determine the factors predicting insulin requirement during GDM. In the present study, we clearly demonstrated that family history of diabetes can be a strong prenatal factor for predicting the insulin requirement in GDM. We also showed that more obese women with higher fasting glucose levels during OGTT and higher HbA1c levels at diagnosis required insulin more often. Moreover, fasting glucose level during OGTT and HbA1C at diagnosis were determined as independent predictors of insulin requirement in GDM. A threshold level of ≥ 89.5 mg/dL in fasting glucose during OGTT or ≥ 5.485% in HbA1C at diagnosis were found to distinguish the cases who required insulin in addition to MNT.

Risk factors that indicate the introduction of insulin therapy for glycemic control in pregnancies complicated by GDM have been studied before. However, a few studies have addressed the predictive value of these factors in these subjects. Some of them have demonstrated the impact of fasting plasma glucose during OGTT on insulin requirement in GDM. A study has shown that women with fasting glucose at or below 95 mg/dL can achieve good glycemic control only after 2 weeks of MNT [17]. Another study has suggested that a fasting glucose level of 87 mg/dL can effectively predict the need for insulin with a sensitivity of 89.1% when compared with the 1- or 2 h values on 75 g OGTT during pregnancy [18]. Langer [9] has found strong positive correlation between fasting glucose above 105mg/dl and maternal-perinatal complications, such as; fetal macrosomia, neonatal hypoglycemia, hypertensive syndromes and cesarean delivery. They have concluded that fasting glucose levels above this cut-off are indicative for starting insulin therapy. Additionally, they report that only 70% of pregnant women with fasting glucose below 95 mg/dL can achieve satisfactory glycemic control only with diet programs. Akinci and colleguages have also demonstrated statistically significant correlation between fasting glucose levels above 105 mg/dL and AIT in GDM [19]. The data given in details above is in accordance with our findings and indicates fasting glucose as a potent predictor of antenatal insulin treatment in GDM. However, different cut-off values may be related to methodology of studies, various target glucose levels or ethnic differences.

Like fasting glucose levels on OGTT, Glycated hemoglobin (HbA1C) has been clearly shown to be useful for prediction of insulin requirement and fetal growth in pregnant cases [15]. In our study, HbA1C predicted poor glycemic control in GDM as reported by Gonzalez-Quintero and Sapienza [10, 11]. More recently, Clayton and co-workers have demonstrated that higher HbA1C, fasting blood glucose, and BMI values are related with AIT in GDM. The use of clinical markers to assess glycemic control early in pregnancy may lead to the earlier identification of women at risk for GDM and earlier introduction of interventions that can decrease the risk for complications [12].

Women with GDM have been demonstrated to have defects both in insulin sensitivity and insulin secretion [20]. The predictive value of fasting glucose for insulin requirement, which is also shown in our study, suggests that the impact of defect in basal insulin secretion is more obvious. Accordingly, Di Cianni et al [21] have shown that women with one abnormal glucose level at fasting on OGTT are characterized with impairment in basal insulin secretory capacity. On the other hand, HbA1c reflects a longer duration of glycemic control and appears to be useful when performed periconceptionally to estimate the risk of congenital anomalies. It does not seem to confer substantial benefit for estimating the risk of fetal overgrowth or other adverse pregnancy outcomes [22]. However, it may be used as a potent predictor of insulin requirement in women GDM, as demonstrated in the present study.

In this study, the positive predictive value of fasting glucose level ≥ 89.5 mg/dL during OGTT and HbA1C level ≥ 5.485% at diagnosis were found to be 73% and 68%, respectively. These relatively low percentages can be considered as weak for being specific predictors for AIT in GDM and they may be attributed to the following factors: The retrospective design of our study is a limitation for controlling the dietary compliance of the participants. Besides, unmeasured fetal or placental factors that influence insulin resistance may have a greater impact on antenatal insulin treament. Finally, self-monitoring capillary glucose measurements were used and decision of introduction of insulin was given accordingly. Kestila and co-workers have shown that continuous glucose monitoring system detects a markedly higher proportion of GDM mothers needing antihyperglycemic medication compared with self-monitoring of plasma glucose [23]. However, daily self-monitoring of blood glucose is useful in gestational diabetes, as suggested at Fifth International Workshop Conference on GDM [5].

In conclusion, considering about the individual differences in severity of glucose intolerance among women with GDM, they should be classified in such a way that will permit the adoption of specific management for each subgroup. Thus, human and financial resources can be applied more appropriately to groups presenting a higher risk of perinatal complications. Our study helps to create awareness of a low and high risk pregnant women for antenatal insulin therapy. Higher fasting plasma glucose during OGTT and HbA1C at diagnosis may better be monitored more closely by specified physicians.
